# Neighborhood Violence Impacts Disease Control and Surveillance: Case Study of Cali, Colombia from 2014 to 2016

**DOI:** 10.3390/ijerph15102144

**Published:** 2018-09-29

**Authors:** Amy R. Krystosik, Andrew Curtis, A. Desiree LaBeaud, Diana M. Dávalos, Robinson Pacheco, Paola Buritica, Álvaro A. Álvarez, Madhav P. Bhatta, Jorge Humberto Rojas Palacios, Mark A. James

**Affiliations:** 1 Department of Pediatrics, Division of Infectious Disease, Stanford University, Grant Building, S 374, 300 Pasteur Drive, Stanford, CA 94305-5208, USA; dlabeaud@stanford.edu; 2 Department of Biostatistics, Environmental Health Sciences, and Epidemiology, College of Public Health, Kent State University, Kent, OH 44240, USA; mbhatta@kent.edu (M.P.B.); mjames22@kent.edu (M.A.J.); 3 Department of Geography, the GIS, Health & Hazards Lab, Kent State University, Kent, OH 44240, USA; acurti13@kent.edu; 4 Department of Public Health and Community Medicine, Universidad Icesi, Cali 760031, Colombia; dmdavalos@icesi.edu.co (D.M.D.); robinson.pacheco.73@gmail.com (R.P.); 5 Grupo de Investigación en Epidemiología y Servicios, Universidad Libre, Cali 760031, Colombia; p3107@hotmail.com; 6 Caucaseco Scientific Research Center, Cali 760031, Colombia; alvaro1@stanford.edu; 7 Stanford University School of Medicine Research IRT, 3172 Porter Drive, Stanford, CA 94305-5208, USA; 8 Secretaría de Salud de Cali, Colombia, Cali 760031, Colombia; jorge.rojas@cali.gov.co

**Keywords:** arboviral surveillance, neighborhood violence, clinical diagnosis, laboratory specificity, spatial clustering, community health

## Abstract

Arboviruses are responsible for a large burden of disease globally and are thus subject to intense epidemiological scrutiny. However, a variable notably absent from most epidemiological analyses has been the impact of violence on arboviral transmission and surveillance. Violence impedes surveillance and delivery of health and preventative services and affects an individual’s health-related behaviors when survival takes priority. Moreover, low and middle-income countries bear a disproportionately high burden of violence and related health outcomes, including vector borne diseases. To better understand the epidemiology of arboviral outbreaks in Cali, Colombia, we georeferenced chikungunya (CHIKV), dengue (DENV), and Zika (ZIKV) viral cases from The National System of Surveillance in Public Health between October 2014 and April 2016. We extracted homicide data from the municipal monthly reports and kernel density of homicide distribution from IdeasPaz. Crucially, an overall higher risk of homicide is associated with increased risk of reported DENV, lower rates of acute testing, and higher rates of lab versus clinical discordance. In the context of high violence as a potential barrier to access to preventive health services, a community approach to improve health and peace should be considered.

## 1. Introduction

Dengue (DENV), chikungunya (CHIKV), and Zika (ZIKV) viruses are examples of vector-borne viruses (arboviruses) transmitted by infected *Aedes aegypti* mosquitoes. Arboviruses are responsible for a significant burden of disease globally [1,2,3], with at least 35% of the population at risk for DENV infection alone [2,3,4]. Though difficult to quantify due to limited data, one estimate attributes 300,000–5,000,000 DALYs (nondiscounted, unweighted disability-adjusted life years) in 2005 to four arboviruses: YFV, Japanese encephalitis virus, Rift Valley fever virus, and CHIKV (before emergence in the Americas) [1]. DENV alone is estimated to be responsible for 390 million infections per year, including 96 million symptomatic cases with 14% of the burden in the Americas [2]. Billions live in areas at risk of these infections [3,5,6,7], which cause acute febrile disease and long-term sequelae [1,8,9,10,11,12]. The long-term impacts of ZIKV are still unclear, as the severe complications at birth for exposed fetuses is likely to be only the tip of the iceberg as epidemiological studies follow these ZIKV-exposed infants through neurodevelopment [13,14,15]. Arboviral outbreaks are forecasted to change and expand in geographic and absolute burden.

Arboviruses cause a public health burden in Colombia. CHIKV emerged as an outbreak between September 2014 and 25 September 2015 [16]. Per the Colombian Ministry of Health and Social Protection Epidemiology Bulletin (25 October–31 October 2015) [17], 712 municipalities reported 439,000 cases during the outbreak [16]. The outbreak disproportionally affected the city of Santiago de Cali which reported 44,877 cumulative cases of apparent disease through 17 October 2015 [18]. DENV is hyperendemic in Colombia and Cali with over 18,000 cases reported to The National System of Surveillance in Public Health (SIVIGILA) in the department of Valle del Cauca between week 1 and week 48, 2015 [17]. ZIKV entered Colombia via Brazil, and the first new case was reported in October 2015 [19]. Between week 40 of 2015 and week 3 of 2016, municipalities reported 20,297 suspected and 1050 lab-confirmed cases to the National Institute of Health [17]. Through April 2016, clinics reported 3139 suspected cases of ZIKV disease to the Municipal Secretary of Public Health of Cali [17].

Violence can impact health delivery-impeding surveillance and delivery of services [20,21,22]—and affect an individual’s health-related behaviors, when survival takes priority [23]. Furthermore, The World Health Organization (WHO) identified violence as a risk factor for increased communicable disease [20]. Violence has previously been studied as a contagious disease exhibiting spatiotemporal clustering and self-prorogation [24,25,26,27,28]. We hypothesize that neighborhood safety and arboviruses are linked in Cali as high rates of violence may act as a barrier to services, including reliable water, mosquito spraying, public health, and epidemiological intervention.

Violence is a complex construct with multiple contributors. Due to lack of more granular data, we use homicide rates as a proxy for the burden of violence. However, violence can have different sources, from individual variables to social, political, and institutional ones, including domestic violence, political violence, politically or socio-economically driven homicides, gangs, microtrafficking, and poverty provoked violence. Social determinants of health such a wealth, education, and violence contribute to health inequities [29,30]. Particularly relevant to the case study in Colombia is political violence. Globally, Colombia is the country with the second most internally displaced persons (IDP), with 7 million (14.5% of the population) between 1985 and August 2017, and 487,129 IDPs between January 2015 and December 2017. Seventy-seven to seventy-eight percent [31,32] of all internally displaced persons live in 234 to 282 of 1122 municipalities. The United Nations Office for the Coordination of Humanitarian Affairs organized these municipalities into departments by priority of most affected areas—Valle del Cauca ranks 12th. Grajales et al. report land control and forced displacement are associated with economic and political movements, creating rural violence and large refugee populations [33].

A review on violence and health in low and middle-income settings (LMIC) highlighted the disproportionate burden violence has on LMICs where over 90% of violence-related deaths occur and the associated mortality rate is nearly 2.5× greater compared to high income countries [34]. Cali is one of the 30 most violent cities worldwide [35], reporting 64 homicides per 1,000,000 people annually. Homicide was ranked as the primary and secondary cause of death in 2008–2014 and 2015, respectively [36]. In 2010, the Ministry of Social Protection prioritized violence as the country’s main public health problem due to its magnitude and impact on health [37]. As noted in a recent pediatric trauma study in Cali, there is a “long-standing history of violence associated with the weak social structure resulting from years of drug trafficking, migration to urban areas, poor economic development, the presence of illegal armed groups, and changed familial, social, and religious patterns” [38].

The municipal secretary of health of Cali publishes an annual action plan which includes activities to prevent both vector-borne diseases and violence; with the caveat that these programs are linked to funding at the city level which are subject to change. Vector-borne disease prevention focuses on breeding site control, community education, and quality health service [39] with the goal of strengthening public health and intelligence surveillance for the integrated analysis of environmental monitoring of the disease; includes surveillance and analysis of morbidity and mortality, etiological agents, risk factors, entomology, reservoirs, and wild populations, to predict, target, and stratify risk [40]. The 2018 plan aims to (1) perform 11,600 drain inspections; (2) decrease arboviral incidence by 10% in priority areas; (3) report 100% of diagnoses confirmed cases of febrile DENV, CHIKV, and ZIKV; and (4) install *Wolbachia* infected *Aedes aegypti* nurseries in 1000 homes [40].

The Municipal 10-year plan for public health focuses violence prevention on strengthening coordination for joint intervention of all government agencies and community empowerment for sexual violence prevention [39]. The Municipal Secretary of Health of Cali (SOH) 2018 action plan includes multiple activities to address violence through community centers for life, harm reduction, promotion of mental health awareness, community-based surveillance and intervention, strengthening mental health help-line, and care for female violence victims [40].

Different geospatial approaches have been used to examine vector-borne diseases at different scales [41,42,43]—from using remote sensing and spatial analysis, to identifying regional/national patterns [44,45,46], to using more localized spatial analysis [47]. Typically, spatial exploratory analysis can be used to identify patterns in both surveillance and potential exploratory variables. DENV geospatial analyses have included either mosquito intensity or human case data [48,49,50]. The hypothesis proposed here was developed from a series of spatial narratives describing local-vector-borne-disease risk in which interrelated topics of poverty, lack of city services, and perceived and actual danger often intersected [50]. Herein, we will analyze patterns of arboviral risk and access to laboratory diagnostics and at the same time take a first step in revealing how arboviral risk is affected by violence by comparing and contrasting arboviral-risk patterns with measures of neighborhood violence.

## 2. Materials and Methods

### 2.1. Ethics

Appropriate local (Universidad Icesi, Cali, Colombia #061) and University institutional review boards (Kent State University #15-529) approved this study. Secondary human case data are presented at the aggregate neighborhood level. As individuals are anonymous, the IRBs deemed informed consent unnecessary.

### 2.2. Location and Study Population

Cali is 160 km from the Pacific coast of Colombia in the department of Valle del Cauca with a population of 2,369,821 in 2015 [36]. The climate is tropical (25.5 °C median temperature and 752 mm annual precipitation from 2015 to 2016) [36]. National annual median GNI per capita is US $7560 [51] with 16.5% of the population in Cali living in poverty and 3.4% living in extreme poverty in 2015 [36]. A child born in Cali in 2015 can expect to live 74.4 years on average [36]. The top two primary causes of death have been homicide and hypertension since 2008 [36]. Populations are constantly immigrating to Cali from all over the country, especially from the regions of Pacific, Cauca, and the coffee growing regions known as the coffee axis. More recently, immigration from Venezuela to Colombia has increased with over one million immigrants registered in just two years including over 16,000 Venezuelans registered as living in the department of Valle del Cauca during the 2018 census as reported the local newspaper and reports from the Ministry of foreign relations in 2018 [52,53,54]. This large-scale immigration could affect transmission dynamics [55,56]. A small proportion (5.3%) of the population of Cali lives in unplanned urbanizations according to a 2015 survey done by the Territorial Organization Plan (POT) [57]. The study population included incident cases of confirmed or suspected CHIKV, DENV, or ZIKV infections from the municipal area of Cali reported between October 2014 and April 2016 to SIVIGILA. Laboratory confirmation was not available for reported Zika cases or chikungunya cases.

### 2.3. Study Design

**Figure A1:**
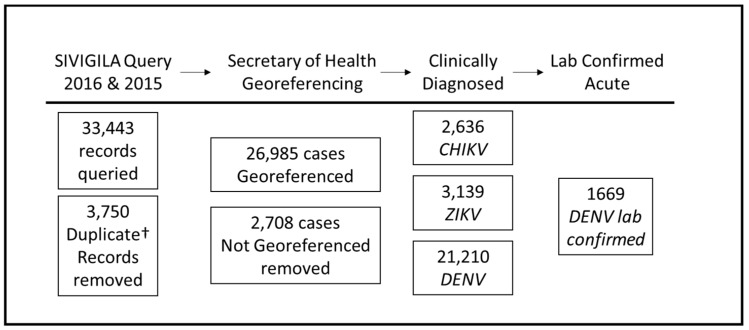
We officially requested the Municipal Secretary of Health to provide cases matching event codes 210 (DENV), 220 (severe DENV), 580 (DENV death), and 217 (CHIKV) for 2014 to 2015. We repeated the query in 2016 to request updated data including event code 895 (ZIKV). † Duplicates defined as same identification number, week of identification, and disease code.

Incident case data were collected retrospectively from The National System of Surveillance in Public Health (SIVIGILA) via the Municipal Secretary of Public Health of Cali. DENV, CHIKV, and ZIKV infections case reports included laboratory results and patient home and work addresses (Figure A1). Three-thousand-seven-hundred-and-fifty duplicate records were excluded from 33,443 initial results.

### 2.4. Data Sources and Linking Data

The following data sources were used to extract data for the final analysis. SIVIGILA. National Institutes of Health of Colombia (INS) maintain ongoing passive, national surveillance in Colombia using SIVIGILA, the agency responsible for the systematic and constant observation and analysis of health events. It is Colombia’s reportable disease database to which all secretaries of health contribute. Rodriguez-Morales et al. [58] previously described the surveillance system. Briefly, in the present study, cases were georeferenced at the neighborhood level according to reported home addresses. DANE. The administrative department of planning of the municipality of Cali publishes the neighborhood level characteristics (2005 DANE Census). We downloaded these reports and compiled the neighborhood level data into comma-separated value (CSV) files. These were linked to shapefiles of Cali neighborhoods and to georeferenced cases by neighborhood. Ideaspaz. As neighborhood level data were not available in the official reports of homicide in Cali, we accessed kernel density files of five levels of homicide risk over space as published by IdeasPaz. We extracted these estimates of homicide risk on 5 May 2018 from JSON (JavaScript Object Notation) files. To validate the smoothed homicide-risk data, we compared the files to comuna-level risk as published by the Inter-institutional Committee on Deaths from External Causes. We mapped the Ideaspaz homicide kernel density layers in ArcGIS^®^ software by Esri (Esri ArcGIS desktop: release 10.3.1, Redlands, CA, USA). The methods to collect the data and construct the kernel density layers are available at www.ideaspaz.org. Briefly, homicide data were extracted from the Ministry of Defense between 1990 and 2016 at the municipal and departmental level. Population estimates and projections were extracted from DANE from 1985 to 2020 at municipal and departmental level. Homicides rate per 100,000 persons was calculated by Fundación Ideas para La Paz at municipal level with data from the DANE population (April 2017) and total homicide cases per municipality (mindefensa). Homicide concentration was estimated by kernel density analysis, with a 100 m edge cell size and a 500 m search radius. Concentration of homicides was extracted from SIEDCO national police data. Inter-institutional Committee on Deaths from External Causes. Due to the lack of temporal data in the kernel density files of homicide risk from IdeasPaz, we also extracted comuna-level-aggregated monthly counts of homicides from official monthly reports by the Inter-institutional Committee on Deaths from External Causes—Observatorio De Seguridad De Cali (http://www.cali.gov.co/). Linking data. Data were extracted from various data sources at the neighborhood level including: education, income, marital status, race/ethnicity, age structure, population, utility services coverage rates, and homicides. The urban Cali shapefile was obtained from the Caucaseco Scientific Research Center. All data were merged with the shapefile at the neighborhood level. Case home addresses were standardized using the SoH guidelines and georeferenced the addresses using municipal secretary of health of Cali software (Supplementary Materials “Estandar creacion archivo para Georreferenciar”).

### 2.5. Case Definitions

ZIKV. Rodriguez-Morales, Galindo-Marquez, García-Loaiza, Sabogal-Roman, Marin-Loaiza, Ayala, Lagos-Grisales, Lozada-Riascos, Parra-Valencia, Rojas-Palacios, López, López, and Grobusch [58] previously described the ZIKV case definition. Briefly, determination of ZIKV infection included either laboratory or syndromic surveillance—clinical definition of fever, rash, conjunctivitis, and arthralgias in a municipality with previous ZIKV circulation, at least one case confirmed by Real-Time PCR (polymerase chain reaction) to detect virus. The clinical definition has been recommended by the World Health Organization (WHO), Pan American Health Organization (PAHO), as well the U.S. Centers for Disease Control and Prevention (CDC). After one case is confirmed by RT-PCR in a municipality, nonrisk patients may be diagnosed by clinical definition [58]. DENV. Villar et al. [59] previously described the DENV surveillance system in Colombia. Briefly, probable and confirmed cases of DENV are reported weekly, and cases of serious dengue disease and mortality due to dengue disease are notified immediately by mandatory reporting to SIVIGILA. In the case of an outbreak, serological samples are taken from 5% of cases of DENV fever (DF) and all cases of serious dengue disease [59]. Sarti et al. [60] previously described the SIVIGILA DENV reports of cases diagnosed as probable and confirmed cases of DF, DENV with alarm signs (DWS), and severe DENV (SD) as defined by the WHO in 2009. Briefly, laboratory confirmation requires anti-DENV IgM and IgG detection, virus isolation, or detection of DENV virus genomic sequences with RT-PCR. Virus isolation is attempted in 6 to 7% of samples received [60]. CHIKV. Determination of CHIKV infection included either laboratory and syndromic surveillance. The clinical definition has been recommended by WHO, PAHO, as well CDC. DENV laboratory diagnostics are reported per SIVIGILA guidelines with acute infection measured by PCR, IgM, or NS1. Test, lab result, and agent were queried to determine testing by acute methods (NS1 ELISA, IgG, PCR, and viral isolation), the result of the assay (Negative, Positive, Equivocal, or No Data), and the agent used in the assay (DENV vs. other). Data were analyzed using apparent disease including both suspected and clinically confirmed (clinical diagnosis, regardless of laboratory confirmation) and laboratory confirmed cases using the definitions provided by SIVIGLA.

### 2.6. Analysis

Kernel density layers of total homicides from Ideaspaz JSON files were mapped in ArcGIS^®^ software by Esri (Esri ArcGIS desktop: release 10.3.1; Redlands, CA, USA). Concentration of homicides was extracted by city from Fundación Ideas Para La Paz from SIEDCO national police data during February 2017. Spatial density was calculated by kernel density analysis using a 100 m edge cell size and a 500 m search radius.

Homicide time series of Inter-institutional Committee on Deaths from External Causes were analyzed using R-Studio, Version 1.0.136 package “Forecast” using an Auto Regressive Integrated Moving Average model (ARIMA) selecting the best model by comparing AICc with varying parameters (*p*, *d*, and *q*) where *p* is the order (time lags), *d* is the degrees of differencing (times the past values are subtracted from the data), and *q* is the order of the moving-average model. The model permitted “drift” or nonstationary seasonality, meaning adjacent seasons moved separately over time. The autoARIMA function estimated the data fit best to 4 time-lags, 1 degree of differencing, and 0 moving average model.

Georeferenced cases of reported DENV, DENV laboratory diagnostics, and confirmed acute DENV density from SIVIGILA were mapped using WGS1984 projection in R Studio, Version 1.0.136 [61]. Spatial density was calculated by kernel density analysis using a 100m edge cell size and a 500 m search radius. Spatial scan statistics were conducted using SatScan [62] with Bernoulli distribution [63]. SatScan searched for high clusters of reported DENV cases, DENV laboratory diagnostics, and confirmed acute DENV using a maximum spatial cluster size of 50 percent of population at risk and a circular window shape. Only secondary clusters without geographic overlap are reported. Clusters were mapped using ArcGIS^®^ software by ESRI (ESRI ArcGIS Desktop: release 10.3.1; Redlands, CA, USA) (Figure 3). The associated relative risk and *p*-value of the hotspot was estimated comparing the ratio of the observed and expected cases under a random distribution as previously described [64].

Generalized liner models (GLM) from R-studio package “stats” were used to estimate adjusted effects of neighborhood relative risk of homicide (IdeasPaz) on number of reported DENV cases (Poisson distribution), low or high levels acute testing (binomial distribution), and low or high levels of confirmed acute illness (binomial distribution). Covariates included neighborhood level social strata, observed number of cases (in the case of acute testing and confirmed acute illness) and population density.

## 3. Results

The SIVIGILA query resulted in 33,443 records; 3750 duplicates were removed if the person ID matched within one week of report for the same disease code; 26,985 cases were georeferenced and 2708 were not located and removed. The final analysis included 2636 CHIKV, 3139 ZIKV, and 21,210 DENV cases (Figure A1).

Of cases reported to SIVIGILA during the study period, the lab confirmed few cases with DENV antigen (Appendix A, Table A1). Of note, we excluded from this analysis: unidentified antigens (*n* = 9) and lab assays omitted from the DENV case reporting form (code: 11, 17, 20, 58, 85, JA, MO, LA) (*n* = 1447). Laboratories confirmed 14% of DENV cases (2989/21,210) by acute methods (PCR, NS1 ELISA, or IgM ELISA), of which only 56.5% tested positive. Acute testing achieved 56.5%, 85.7%, 56.1%, and 57.1% sensitivity compared to clinical diagnosis of severe DENV fever, nonsevere DENV fever, and DENV death respectively (Table 1).

**Figure 1:**
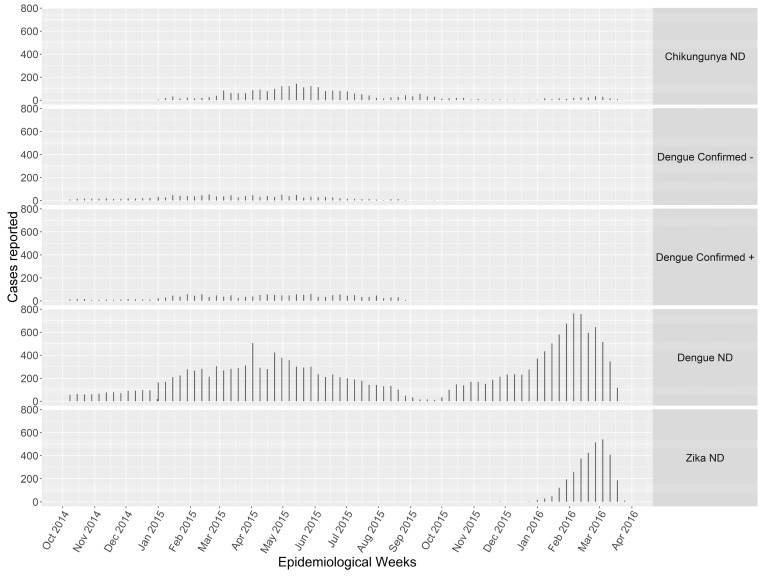
Cases reported to The National System of Surveillance in Public Health (SIVIGILA) over time. Laboratory results reported as per SIVIGILA. Acute diagnostics included PCR, IgM, or NS1. We queried the variables test, lab result, and agent to determine acute methods (NS1 ELISA, IgG, PCR, and viral isolation), the result of the assay (Negative (−), Positive (+), Equivocal, or No Data (ND)), and the agent used in the assay (DENV vs. non). NA = data unavailable from lab.

Patterns in incident arboviral cases emerged over time with clear outbreak seasons (Figure 1) and concurrent outbreaks. There appears to be either a real increase in cases over the epidemic curve or a tendency to diagnose what is in an outbreak as sensitivity remains constant (interquartile range = 0.4–0.6) over time (Figure 1).

**Figure 2:**
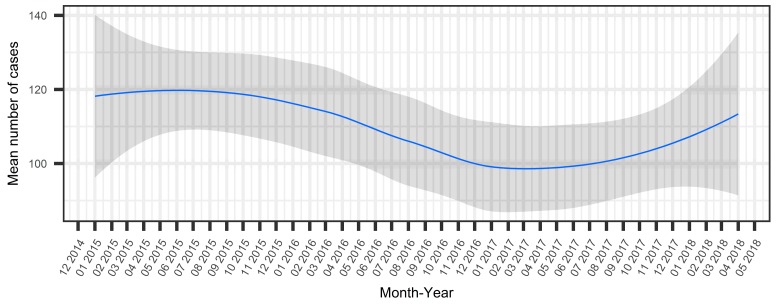
Loess smoothed mean and 95% CI of homicides reported by month (Inter-institutional Committee on Deaths from External Causes monthly reports).

**Figure A2:**
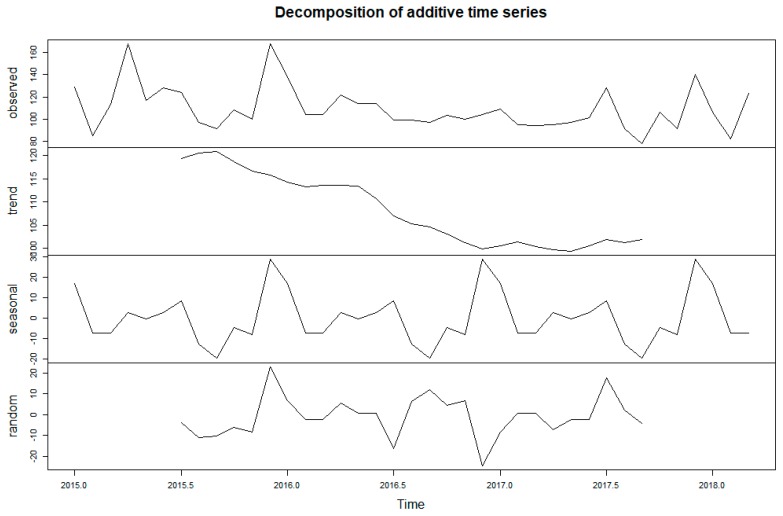
Decomposition of time series trends in monthly homicide reports. Panel observed shows observed time series data. Panel “trend” shows general trend of decreasing monthly homicides between January 2015 and March 2018 (monthly homicides = −16.07ln(x) + 154.84; *R²* = 0.9119). Panel “seasonal” shows a seasonal trend with marked peaks near the beginning of the year and a smaller peak at mid-year. Panel “random” shows the unexplained variation.

Homicide rates varied over time (Figure 2) (Pearson’s correlation coefficient = 0.37 95% CI = 0.3–0.4, *p*-value < 0.0001). By decomposing the time series using the R “forecast” package (Appendix A, Figure A2), we visualized the observed time series, trend, seasonal effects, and random effects (Supplementary Materials).

Average monthly homicide risk rates varied by region with the lowest (1.38 cases) and highest (12.71) risks reported in Comunas 22 and 15, respectively.

**Figure 3:**
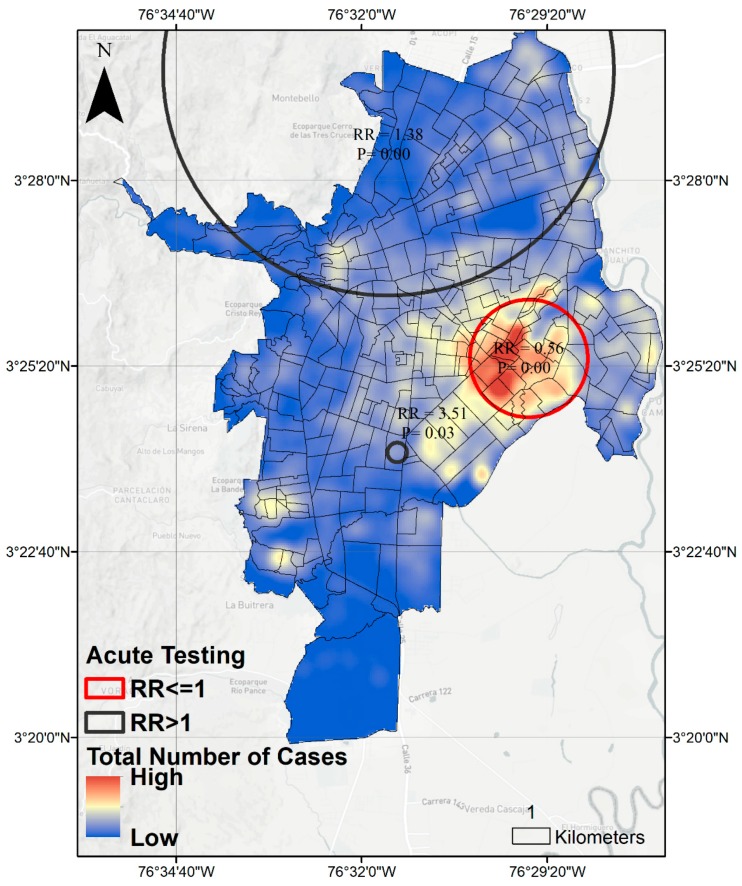
SatScan analysis of acute DENV laboratory testing among all DENV cases reported to SIVIGILA in Cali 2014–2016. RR = Relative Risk calculated as ratio of observed versus expected number of cases having lab confirmation of acute infection compared to no lab confirmation or non-acute lab diagnosis in urban area of Cali, Colombia.

In spatial analysis, we observe spatial clustering of acute testing of DENV among all DENV cases reported. Likelihood of lab confirmed acute infection increased in the Northwest region of the city compared to other regions (relative risk = 1.38, *p* < 0.001) and decreased in the central-eastern region (RR = 0.56, *p* < 0.001), also reporting higher total numbers of DENV cases (Figure 3).

**Figure 4:**
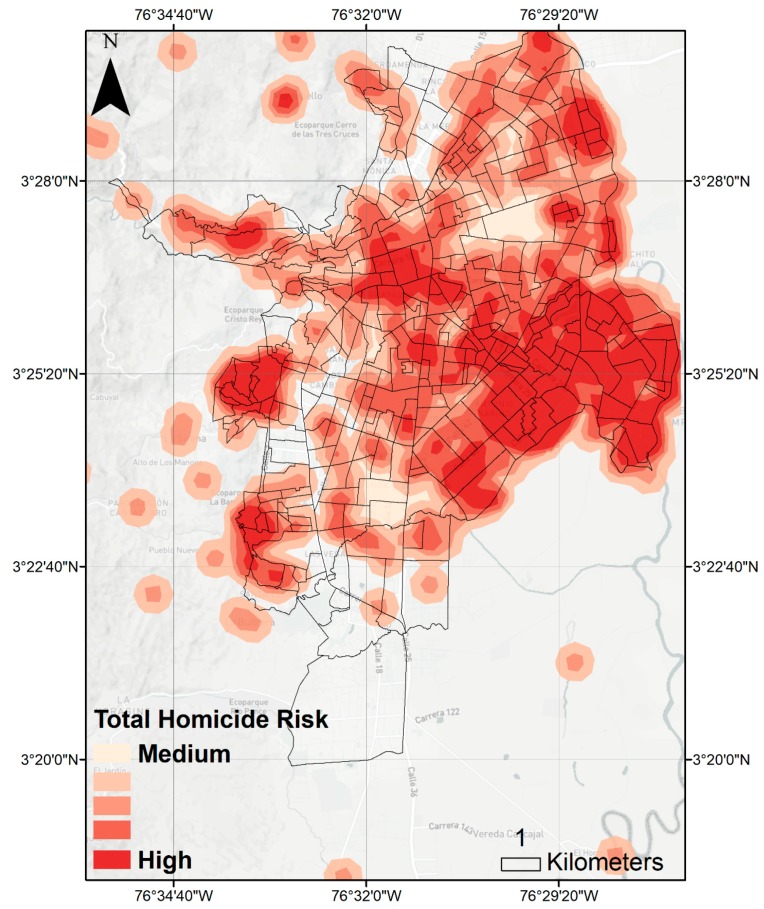
Total homicide risk kernel density in urban area of Cali, Colombia. Extracted from IdeasPaz on 5 May 2018. Briefly, homicide data were extracted from the Ministry of Defense between 1990 and 2016. Homicide concentration was estimated by kernel density analysis, with a 100 m edge cell size and a 500 m search radius. Concentration of homicides was extracted from SIEDCO national police data.

We observed spatial clustering of homicides-homicides increased in the central-east of the city compared to other regions. This region overlaps with the high DENV risk and low acute testing (Figure 4).

**Figure 5:**
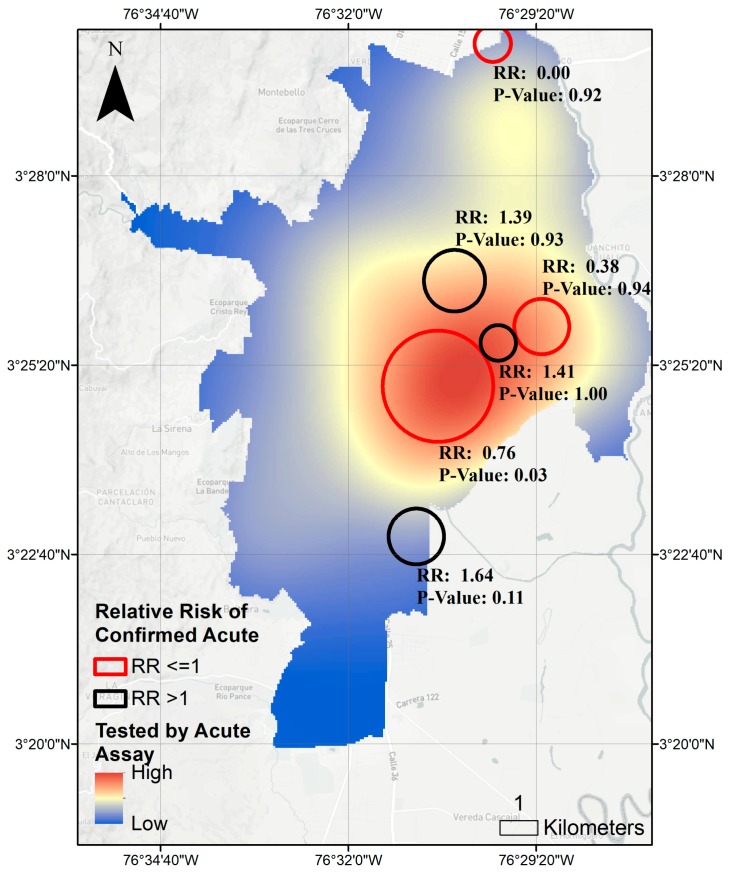
Spatial clustering of true DENV positives among acutely tested DENV cases reported to SIVIGILA in urban area of Cali, Colombia 2014–2016. RR = Relative Risk calculated as ratio of observed versus expected number of cases having lab a positive lab results compared to negative lab result among cases tested by lab for acute DENV infection in urban area of Cali, Colombia.

Among acutely tested cases, we observed spatial clustering of sensitivity of lab diagnostics compared to clinical diagnosis (Figure 5).

Lower median neighborhood social strata (government assessed level of median neighborhood income level) and higher homicide risk is associated with higher burden of DENV (*p* < 0.001, Table 2, Absolute Burden of DENV); lower rates of acute testing (*p* < 0.001, Table 2, Access to DENV Laboratory Testing); and higher rates of discordance between lab diagnosis and clinically suspected DENV cases (*p* < 0.001, Table 2, Discordance Between Lab and Clinician).

Controlling for SES and population density, overall kernel density of reported DENV risk (suspected and confirmed acute) is associated with increased risk of homicide and increasing wealth (social strata) (Table 3, GLM Poisson Regression for Kernel Density of DENV RISK (1–5)). Controlling for absolute burden of observed cases, SES, and population density, access to access to DENV laboratory testing decreased with increasing homicide risk and decreasing wealth (*p* < 0.001) (Table 3, GLM Binomial Regression for Acute Testing RR above and below 1).

## 4. Discussion

We observe an intersection between violence and health—risk of arboviral infection and homicide are geographically clustered, specifically in the central-western region. These findings support our hypothesis that reported violence impact disease risk. Amongst clusters of limited access to acute testing, we found an association with lower social strata and increased homicide risk. We observe low sensitivity of lab-diagnostics (compared to clinical diagnosis) associated with lower social strata and higher homicide risk. However, as with other studies exploring fine-scale relationships between disease and violence [65], data deficiencies limit sophisticated modeling. In Cali, these deficiencies are likely under-reported in disease surveillance and unavailable or context-poor spatial data on violence.

A WHO violence and health report [20] identified violence as a risk factor for increased communicable disease and identified specific factors increasing risk of communicable during conflicts: (1) the decline in immunization coverage; (2) population movements and overcrowding in refugee camps; (3) greater exposure to vectors and environmental hazards, such as polluted water; (4) the reduction in public health campaigns and outreach activities; and (5) the lack of access to health care services. Furthermore, specific arboviral examples are becoming more apparent [21,22]: one DENV intervention study concluded that the wider social context of urban violence and insecurity hindered intervention acceptance despite the potential for DENV prevention and called for further research on insecurity’s impact on DENV prevention programs [21]. An intervention for mobilizing against *Aedes aegypti* under difficult security conditions in southern Mexico found urban violence inhibited DENV prevention [22]. We previously found that perceived risk factors included proximity to standing water, canals, poverty, invasions, localized violence, and military migration. These risks overlapped arboviral case density maps and identified areas suitable for transmission but are possibly underreporting to the surveillance system [50].

Localized patterns of arboviruses can result from a variety of different ecological, socio-economic, behavioral, and political factors. While studies have previously considered different aspects of this disease system for Cali, a notably absent variable in these epidemiological analyses has been the impact of violence. While the interrelationship between disease presence and safety/security is likely to be a factor in most environments, this is especially pertinent for Cali, with its well-documented legacy of violence. In this paper we have made the first step in linking disease presence and violence spatially and temporally by considering the co-occurrence of arboviral risk and homicide risk. Unfortunately, the quality and scale of violence-associated data lags other more traditional arbovirus surveillance data, meaning our findings are hypothesis generating, especially at finer subneighborhood scales where results could influence vector control strategies.

Gaps in arboviral surveillance system exist in this region, where cases of arboviral infection are routinely under-reported due to complex social, economic, and political factors [50], including violence. Use of health services such as laboratory diagnostics, and subsequent case reporting, is influenced by access and the external environment. Access defined as the fit between the patient and the health care system, is determined by five factors: availability, accessibility, accommodations, affordability, and acceptability [66]. Increased access and decreased cost are expected to lead to increased formal care treatment-seeking behavior, and thus increased positive outcome. The question remains as to how violence affects accessibility, for example, in terms of clinic location, hours of access, and perceived safety in attending. We suspect systematic underreporting by region according to access to health services related to SES as previously reported by Sarti et al. [60]. Access to preventative services may be inversely related to violence, as previously noted [21,22]. An independent study reported aggregated-confirmed-DENV incidence rates 5.8× and 3.5× higher compared to Colombian state and local levels, respectively [60].

We observed spatial clustering of lab-confirmed acute infection (Figure 5), suggesting underlying geographic patterns in the access to DENV-laboratory testing. One study in Kenya [67] found access to care varies by the level of care available locally. Furthermore, we observed spatial clustering of specificity of clinical diagnosis compared to lab-confirmed acute cases (Figure 5), suggesting variable quality of clinical care. These clusters are correlated with social strata of the neighborhoods in the clusters (Table 2), after controlling for absolute arboviral burden.

Overall, laboratory confirmation rates of arboviruses are low (Table 1). The Special Programme for Research and Training in Tropical Diseases, with researchers and representatives from ministries of health found low lab confirmation rates [68], confirmed by others [69], which limits the surveillance system, response time, and outbreak response. As reported previously [70,71], clinical diagnosis achieved low specificity compared to lab diagnosis (Table 2). This may impact clinical care. Surveillance systems of asymptomatic arboviral transmission suffer from underreporting and a lack of access to care around the world [72,73]. For example, one study in Brazil estimated 12–17:1 DENV cases per reported case in the community [74], with comparable results observed in Nicaragua, Thailand, Cambodia, Brazil, Colombia, Mexico, and Philippines [60].

We find evidence of concurrent outbreaks of DENV, CHIKV, and ZIKV (Figure 1)—however, laboratory confirmation was only available for DENV cases which may include clinical misdiagnosis [75,76] (Table 1), especially with coinfection [77,78,79,80], and serological cross-reactivity between closely-related flaviviruses DENV and ZIKV [81,82,83,84,85]. Previous studies identified patterns explaining co-endemicity of human DENV surveillance data [50,86,87,88,89,90,91,92,93]. Globally from 1952 to 2017, 49.2% (123/250) of the studied countries/territories reported two or more *Aedes* spp.-transmitted diseases in common [90]. Recent studies have reported DENV, CHIKV, and ZIKV co-circulation in South America [50,91,92,93]. Our work supports previously identified temporal patterns of human DENV infections surveillance; finding clear temporal sequence [94], temporal patterns varying from year to year [95], correlation with seasonal climate [96]; and spatiotemporal patterns [96,97].

The quality of violence-related data limits our ability to co-model the outcomes identified here. Interestingly, homicides also displayed temporal patterns with a trend of decreasing risk from 2015 to 2018 and marked seasonal variation. However, within this decreasing trend, finer-scale patterns emerge. Violence rates remain high in some regions, suggesting underreported disease burdens and lower access to DENV laboratory testing and confirmation. Furthermore, the central-east of Cali reported higher homicide rates on the exterior of the eastern urban boundary, which experiences invasion, or the establishment of informal settlements [57]. Here, for example, an additional risk of mosquito breeding habitats associated with typical informal settlement living is also present. This provides a considerable public health challenge: areas with a high density of informal settlements have high rates of violence yet more limited access to diagnostics and vector control. Moreover, the nature of these settlements is likely to facilitate mosquito breeding. While our data limits our ability to resolve finer-scale spatial patterns, at a more aggregate scale we did find an association between increased risk of reported arboviral infection and reported homicide risk.

Recently, Geographic Information Science is reassessing appropriate spatial-scale of analysis considering both physical and social-behavioral context, interpretation, and public health implications. The uncertain geographic context problem [98,99] states: if data does not capture the human experience, then subsequent analyses and insights will be deficient [100]. Although challenging to collect, context-rich data would improve traditional epidemiological analysis. Vector-borne disease maps usually include environmental (including moisture and vegetation), infrastructure, climatic (micro and macro), entomological, and human data layers (density, social, behavioral, political, and disease surveillance) [101,102]. Public health intervention requires data on subneighborhood scale features, such as houses, streets, standing water, and trash [103,104] which are traditionally incomplete or available at courses space-time scales. For example, while human density, economic hardship [105,106], and standing water are associated with DENV, these relationships may vary in intensity and co-vary to produce microregions of risk. New mixed-methods of data collection are required to quantify and map the context of patterns of violence and disease risk identified here.

The community context of violence permeates private lives. Venegas Luque et al. [23] reviewed the psychosocial and mental health effects of internal violence in Colombia, citing legal actions in which the government takes responsibility for psychosocial care of the displaced population; recognizes how violence effects personal and community stability; reinforces the displaced population’s right to health; emphasizes mental health care; and consolidates public policy for the care and reparation of violence victims [23]. High rates of domestic violence have also been reported in Cali, with 35% of children (5–9 years old) and 31% of women experiencing domestic abuse in 2005, although the authors suspected high rates of underreporting [107]. In the same study, communes 13, 14, and 15 in the District of Aguablanca and commune 6 had the highest concentration of cases of domestic violence [107], the same regions identified in the current study for high arboviral and violence risk.

**Figure 6:**
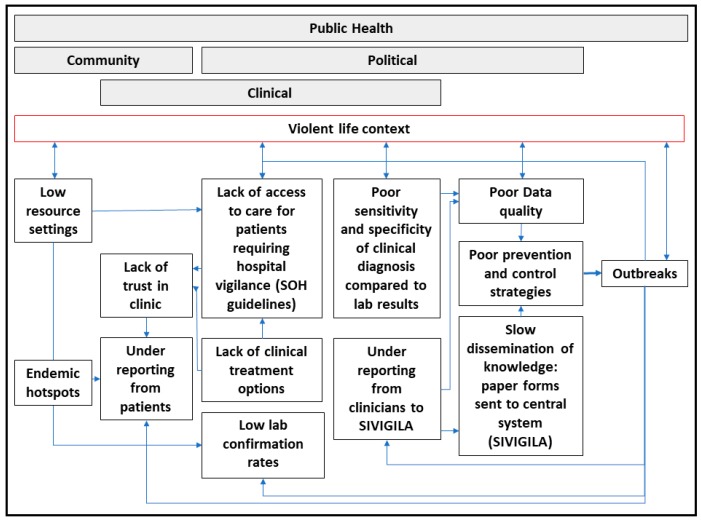
Gaps in current arboviral surveillance system and violence as barrier to preventative health service access.

Some proximate barriers to laboratory diagnostics and case reporting have been suggested: health care access, especially in areas most at-risk of arboviral infection [50]; and/or limited public health resources for data management and reporting (Figure 6). Patients who report to clinics may be excluded from hospital surveillance required by the municipal secretary of health guidelines, placing them at higher risk of mortality (SoH guidelines). Patients living in violent contexts may be especially vulnerable to lack of access to health care services as identified by WHO [20]. Further, the urban poor may be disproportionately affected by outbreaks in the future [56,108,109] as the potential for outbreaks changes with temperature [110,111,112], vector adaptation to urban environments (including water storage and household containers) [113,114], and violence as a barrier to preventative services [20,21,22]. The long-term impacts of arboviral infection, especially in children, could create a vicious cycle of disability, poverty, and violence if left uncontrolled [115,116].

## 5. Conclusions

We present outbreaks of DENV, CHIKV, and ZIKV at the macro- and microscales in Cali and identify patterns of homicide associated with reported cases during an outbreak over time and space. Factors secondary to traditional epidemiological surveillance and public health efforts in this endemic region, such as neighborhood violence, can impact endemic and epidemic disease. These trends could be further analyzed if violence data were available at a finer spatial scale. We have linked arboviral surveillance and violence spatially. We observed spatial clustering of both acute testing and specificity of clinical vs. laboratory diagnosis. An overall higher risk of homicide is associated with increased risk of reported DENV, lower rates of acute testing, and higher rates of lab versus clinical discordance. While we do not suggest that increased homicide rates themselves drive increased mosquito-borne disease rates, the correlation shown here underscores the need for integrated vector control; environmental health; and a community approach to holistically improve public health. New data shows peace-generation may be incentivized using a system similar to carbon credits [117]. Furthermore, the NGO Cure Violence believes that violence can be treated as an infectious disease using a Health Violence Cure Model which is being implemented in over 60 communities in twenty-five U.S. cities and five continents [26] with success rates up to 70% [118].

## Figures and Tables

**Table 1:** Results for acute dengue virus (DENV) testing by clinical severity.

	Nonsevere DENV	Severe DENV	DENV Death	Total
Total acute testing	2904	42	7	2953
Negative	1275 (6%)	6 (5%)	3 (23%)	1284
Positive	1629 (9%)	36 (28%)	4 (46%)	1669
Sensitivity compared to clinical diagnosis	56.1%	85.7%	57.1%	56.5%

**Table 2:** Absolute burden of DENV by social strata and total homicide risk; DENV acute testing rates by social strata and total homicide risk; and confirmed DENV acute by social strata and total homicide risk.

	Absolute Burden of DENV		Access to DENV Laboratory Testing		Discordance Between Lab and Clinician	
	Lower risk of Total DENV	Higher Risk of Total DENV	Higher Rates of Acute DENV Testing	Lower Rates of Acute DENV Testing	Higher Rates of Confirmed Acute DENV	Lower Rates of Confirmed Acute DENV
*n*	19,673	19,672	6185	5561	306	3695
Median Social Strata of Neighborhood (%) *p* < 0.001.						
Low	3176 (16.9)	1997 (10.6)	208 (3.4)	1526 (27.4)	0 (0.0)	378 (10.2)
2	2568 (13.6)	5855 (31.1)	2007 (32.7)	3193 (57.4)	0 (0)	2158 (58.4)
3	1525 (8.1)	8476 (45.0)	3222 (52.5)	842 (15.1)	40 (13.1)	1159 (31.4)
4	1786 (9.5)	1906 (10.1)	204 (3.3)	0 (0.0)	259 (84.6)	0 (0.0)
5	5395 (28.7)	584 (3.1)	414 (6.8)	0 (0.0)	0 (0)	0 (0)
High	4368 (23.2)	0 (0)	66 (1.1)	0 (0.0)	0 (0)	0 (0)
Not reported	-	-	11 (0.2)	0 (0.0)	7 (2.3)	-
Homicide Risk over space (%) *p* < 0.001.						
Low	6522 (34.7)	230 (1.2)	204 (3.3)	0 (0.0)	16 (5.2)	0 (0.0)
1	122 (0.6)	566 (3.0)	128 (2.1)	0 (0.0)	0 (0)	0 (0)
2	3942 (20.9)	2452 (13.0)	1101 (17.8)	0 (0.0)	75 (24.5)	23 (0.6)
3	3446 (18.3)	3688 (19.6)	1750 (28.3)	18 (0.3)	94 (30.7)	449 (12.2)
4	2715 (14.4)	5492 (29.2)	1824 (29.5)	537 (9.7)	121 (39.5)	1422 (38.5)
High	2071 (11)	6390 (34.0)	1178 (19.0)	5006 (90.0)	0 (0)	1801 (48.7)

**Table 3:** DENV cases reported by region by social strata and homicide risk (GLM, Poisson) and GLM binomial regression for acute testing Relative Risk above and below 1.

	GLM Poisson Regression for Kernel Density of DENV RISK (1–5)			GLM Binomial Regression for Acute Testing RR above and below 1		
	OR	2.5%	97.5%	OR	2.5%	97.5%
Homicide Risk	1.14 *	1.13	1.14	0.03 *	0.02	0.04
Social Strata	1.06 *	1.05	1.06	5.18 *	3.89	7.01
Interaction of Social Strata and homicide risk	1.02 *	1.02	1.02			
Population Density	1.11	1.11	1.12	0.95	0.84	1.08
Observed DENV Cases				1.02 *	1.02	1.02

* *p* < 0.001; Homicide risk by spatial join between case acute testing RR and kernel density of homicide risk (1–5). Social strata and population density by spatial join between case acute testing RR and neighborhood mean social strata (1–6) and population density (population/squared meters). OR = Odds Ratio. GLM: Generalize Linear Regression; RR: Relative Risk.

## Supplementary Materials

The following are available online at http://www.mdpi.com/1660-4601/15/10/2144/s1, Neighborhoods by Comunas shape file. Estandar creacion archivo para Georreferenciar.

## Appendix A

**Table A1:** DENV lab results by assay and antigen.

Assay	IgM	PCR	NS1 ELISA	Viral Isolation	Total acute	DENV IgG
DENV (*n* = 21,210)						
Tested (%)	2727 (12.9)	39 (0.18)	216 (1.01)	2 (0.001)	2953 (13.9)	196 (0.9)
Positive (%)	1496 (54.9)	28 (71.8)	145 (67.1)	0 (0)	1669 (56.5)	137 (69.9)
