# Supplementary material for: Neighborhood Violence Impacts Disease Control and Surveillance: Case Study of Cali, Colombia from 2014 to 2016
Source: Int J Environ Res Public Health. 2018 Sep 29;15(10):2144. doi: 10.3390/ijerph15102144 (PMC6211120; doi:10.3390/ijerph15102144)
Supplement: Supplementary file 1 [file ijerph-15-02144-s001.zip › Estandar creacion archivo para Georreferenciar.pdf]

ALCALDÍA DE SANTIAGO DE CALI
DEPARTAMENTO ADMINISTRATIVO DE PLANEACIÓN

IDESC
INFRAESTRUCTURA DE DATOS ESPACIALES DEL MUNICIPIO DE SANTIAGO DE CALI

ESTÁNDAR PARA LA CREACIÓN DE UN ARCHIVO PARA SER GEORREFERENCIADO

A continuación se describen las pautas y la estructura para la generación de un archivo para ser georreferenciado.

1. Crear un archivo en Microsoft Excel.

2. El nombre del archivo de Excel no debe contener espacios en blanco, ni caracteres extraños. (Ej: archivo_01.xls)

3. La primera fila deberá contener los nombres de los campos o atributos y estos no deberán superar los diez (10) caracteres, ni contener caracteres especiales (como tildes, eñes, etc.), ni contener espacios en blanco. Un ejemplo correcto del nombre de un campo es: **campo_01**

4. Crear un campo con el nombre '**cod**', cuyos valores deben ser un consecutivo, lo que permitirá identificar los registros que no puedan ser georreferenciados. (La no georreferenciación es debida, a que la dirección se encontraba mal escrita, o porque el georreferenciador no pudo localizarla o porque se encontraba en zona rural. Estos registros se deberán georreferenciarse de forma manual por el usuario.)

5. El archivo no debe contener ningún tipo de formato como colores, filtros, división de la tabla, etc.

6. El campo que contiene la dirección y el cual permitirá la georreferenciación del archivo, debe llamarse '**direccion**'. (sin tilde)

7. Las direcciones deben estar escritas de acuerdo al siguiente formato:
   AV: Avenida
   CL: Calle
   KR: Carrera
   PJ: Pasaje
   TV: Transversal
   DG: Diagonal
   N: Norte
   O: Oeste

   Para más información ver la guía para la nomenclatura de Cali:
   http://idesc.cali.gov.co/download/guia_nomenclatura_cali.pdf

   Ejemplos:
   - AV 2 # 13 N - 31
   - AV 4 O # 6 O - 170

- AV 2E # 24 N - 10
- CL 5 # 39 - 42
- CL 56A # 42C 2 - 35
- CL 2 O # 22 - 31
- KR 1 # 21 - 36
- KR 38 # 5B - 38
- KR 2 N # 22 - 103
- KR 22 # 2 O - 56
- PJ 7F # 66 - 24
- TV 2A # 1C - 14
- DG 28C # 43A – 41

8. Finalmente el archivo de Excel se debe guardar como un archivo de texto (delimitado por tabulaciones) (*.txt) y enviarlo al correo idesc@cali.gov.co

Ingeniero
Julio A. Muñoz
Coordinador
Infraestructura de Datos Espaciales de Santiago de Cali - IDESC
Alcaldía de Santiago de Cali
Departamento Administrativo de Planeación Municipal

Enviar por Orfeo
